# Error in Byline

**DOI:** 10.1001/jamanetworkopen.2025.46760

**Published:** 2025-11-05

In the Invited Commentary titled “Contemporary Blunt Splenic Injury Management—Less Is More,”^1^ published September 23, 2025, Dr Shehadeh’s name was misspelled in the byline. This article has been corrected.^1^
